# Population Genetic Structure of the Cotton Bollworm *Helicoverpa armigera* (Hübner) (Lepidoptera: Noctuidae) in India as Inferred from EPIC-PCR DNA Markers

**DOI:** 10.1371/journal.pone.0053448

**Published:** 2013-01-11

**Authors:** Gajanan Tryambak Behere, Wee Tek Tay, Derek Alan Russell, Keshav Raj Kranthi, Philip Batterham

**Affiliations:** 1 Department of Genetics, Bio21 Molecular Science and Biotechnology Institute, The University of Melbourne, Parkville, Melbourne, Victoria, Australia; 2 CSIRO Ecosystem Sciences, Canberra, Australian Capital Territory, Australia; 3 Department of Agriculture and Food Systems, The University of Melbourne, Parkville, Melbourne, Victoria, Australia; 4 Division of Entomology, Indian Council of Agricultural Research, Research Complex for North Eastern Hill Region, Shilong, Meghalaya, India; 5 Central Institute for Cotton Research, Nagpur, Maharashtra, India; Natural Resources Canada, Canada

## Abstract

*Helicoverpa armigera* is an important pest of cotton and other agricultural crops in the Old World. Its wide host range, high mobility and fecundity, and the ability to adapt and develop resistance against all common groups of insecticides used for its management have exacerbated its pest status. An understanding of the population genetic structure in *H. armigera* under Indian agricultural conditions will help ascertain gene flow patterns across different agricultural zones. This study inferred the population genetic structure of Indian *H. armigera* using five Exon-Primed Intron-Crossing (EPIC)-PCR markers. Nested alternative EPIC markers detected moderate null allele frequencies (4.3% to 9.4%) in loci used to infer population genetic structure but the apparently genome-wide heterozygote deficit suggests in-breeding or a Wahlund effect rather than a null allele effect. Population genetic analysis of the 26 populations suggested significant genetic differentiation within India but especially in cotton-feeding populations in the 2006–07 cropping season. In contrast, overall pair-wise *F*_ST_ estimates from populations feeding on food crops indicated no significant population substructure irrespective of cropping seasons. A Baysian cluster analysis was used to assign the genetic make-up of individuals to likely membership of population clusters. Some evidence was found for four major clusters with individuals in two populations from cotton in one year (from two populations in northern India) showing especially high homogeneity. Taken as a whole, this study found evidence of population substructure at host crop, temporal and spatial levels in Indian *H. armigera*, without, however, a clear biological rationale for these structures being evident.

## Introduction

The polyphagous nature of the Old World cotton bollworm *Helicoverpa armigera* (Hübner) on a wide range of wild and crop hosts across different ecological zones, its highly variable life-history traits (e.g., number of generations, crop hosts, presence of summer/winter diapause) and seasonal abundance present a unique challenge for ecological and evolutionary studies. The number of generations possible per year is directly influenced by temperature, rainfall and presence of suitable hosts [1]. In India, *H. armigera* is an important pest of cotton, legumes, cereals and vegetables, and presents a unique challenge to those studying its population genetic structure.

The farming landscape in India is predominantly characterised by small farms and mixed cropping systems. The cropping patterns in India normally ensures the presence of five to six different host crops in different proportions for *H. armigera* at any given time of the growing season [2], thereby creating a heterogeneous matrix of hosts which provide ideal platforms for *H. armigera* to move between hosts and geographic areas throughout the year. Furthermore, the presence of three major cropping scenarios in India (in the North, Centre and South) are influenced by the pattern of the monsoons [3] (i.e., southwest monsoons: June to September and northeast monsoons: October to December), and by the sub-tropical nature of the south that allows continuous cropping versus the more continental and temperate climate of the north. India's cropping scenarios therefore provide a range of hosts crops for *H. armigera* all year round in any given region, although cotton represents the main host crop on which this pest species completes three out of possible seven to eight generations annually in 11 states [3], [4], [5], see [6] for a map of cotton states. In the north, facultative pupal diapause is reported in the winter months following the cotton season [7], [8], [9], with synchronous emergence of large numbers of moths frequently triggered by the first heavy rainfall (after the arrival of the monsoon) after prolonged dry periods [10]. The first post-diapause generations in the north are on crops and weeds other than cotton. In the mid-hill regions of Himachal Pradesh in northern India, chickpea is the first crop to be exploited by over-wintered *H. armigera* populations, between March and May [11]. Windborne long-distance migration of *H. armigera* in central India is likely to occur at the end of the cropping season (December–January), while rains prolong the growing season in northern and southern India, with the resulting adult migration in these regions typically occurring around March–April [12]. The temporal pattern of host availability and importance in the agricultural landscape therefore varies in a complex mosaic across India.

Over the past three decades, there has been speculation that Indian *H. armigera* could be categorized into races based on host-feeding preferences and limited inter-mating (e.g. [13], [14]). Such genetic diversity in connection with host plants has been previously shown in *H. armigera* in Australia [15] where there is, for example an identifiable lucerne-preferring ‘race’. Variable metabolic mechanisms mediating pyrethroid resistance have been reported with a shift from mixed-function oxidase-mediated pyrethroid resistance to an esterase-mediated mechanism during mid October in central Indian *H. armigera* populations [16], attributable to both the influx of moths from other populations [17], [18] and the emergence from diapause of moth populations with genetic make-ups different from that of the non-diapausing population [19], [20]. Differential responses to pheromones in different populations and variations in parasitoid responses have been reported [2], [21], and can possibly be interpreted as reflecting an influx of populations between different agricultural systems from different ecological zones, although this view has not yet been tested using population genetics data. Recently, genetically modified (GM) cotton varieties which expressed *Bt-*toxins Cry1Ac and Cry2Ab have made important contributions in reducing application frequencies and dosage of insecticides for the control of *H. armigera*. The intense selection with *Bt* proteins may contribute to population substructure, while evolutionary constraints to host crop preferences may further contribute to area-wide gene flow patterns [22]. All these factors may result in genetic patterning in the species across the Indian agricultural landscape. Understanding the movements of *H. armigera* adults between GM and non-GM crops, or between sprayed and unsprayed crops will be crucial to the management of *Bt* and insecticide resistance in this pest.

The only India-wide major polyphagous crop pest thoroughly examined for genetic diversity is the whitefly *Bemisia tabaci* which comprises a polyphagous species complex with ecological niche separation with respect to host plant (and some geographic) preference [23]. It has at least 6 biotypes in India and probably many more. In particular the older Asia I groupings had a preference for eggplant and Asia II for tobacco and cassava. The more recently introduced B-biotype does particularly well on Tomato which only 1 of the 14 Asia 1 ‘races’ does. This is a particularly complex example but it does show the potential for such separations in other widespread polyphagous species.

Studies of *H. armigera* population genetics based on different DNA markers such as random amplified polymorphic DNA [24], isozymes [25], mtDNA [26] and microsatellites (e.g., [27]; [28]) have been reported. These studies found little genetic variation between widely separated populations, supporting the idea that extensive long distance migration was occurring in *H. armigera*. In Australia, studies have revealed small genetic distances between widely separated populations based on isozymes [29], mitochondrial DNA polymorphisms [30], and sodium channel gene alleles [31]. In contrast, studies of Scott et al. [32], [33], [34], [35] based on microsatellites suggested substantial population substructure in Australian populations of *H. armigera*. Endersby et al. [28] applied markers developed by both Scott et al. [36] and Ji et al. [37] to study Australian *H. armigera* populations collected from the southern and western regions of Australia and found no significant patterns of population substructure. The conflicting findings of Scott et al. [32], [33], [34], [35], Endersby et al. [28] and Weeks et al. [38] were due, at least in part, to factors associated with allele drop-outs (ADO), null alleles caused by mutations at primer annealing sites [28], and microsatellite loci being associated with non-LTR RTE retrotransposable elements (TE's) in Scott et al.'s analyses [39].

Given the wide distribution and migratory ability of *H. armigera*, effective and reliable molecular genetic markers must demonstrate efficiency in PCR amplification in individuals from within and between populations within a country, and between populations from different countries. Although less likely to be affected by TE-induced PCR failures as seen in various lepidopteran microsatellite markers (including three for *H. armigera*, [39]), Exon-Primed Intron-Crossing (EPIC)-PCR markers [40], [41] nevertheless are susceptible to null alleles if exon regions are variable at primer annealing sites, although this is yet to be demonstrated in population genetics studies. This study applies EPIC-PCR markers designed specifically for *H. armigera* [42] to generate data for testing the hypothesis that geographical and host plant components are significant factors underlying genetic variation in Indian *H. armigera*. In the absence of detailed knowledge of gene flow and for the purpose of this study we regard as ‘populations’, samples of *H. armigera* taken from different crops, areas and/or at different times.

## Materials and Methods

### Sampling and DNA extraction

**Figure 1:**
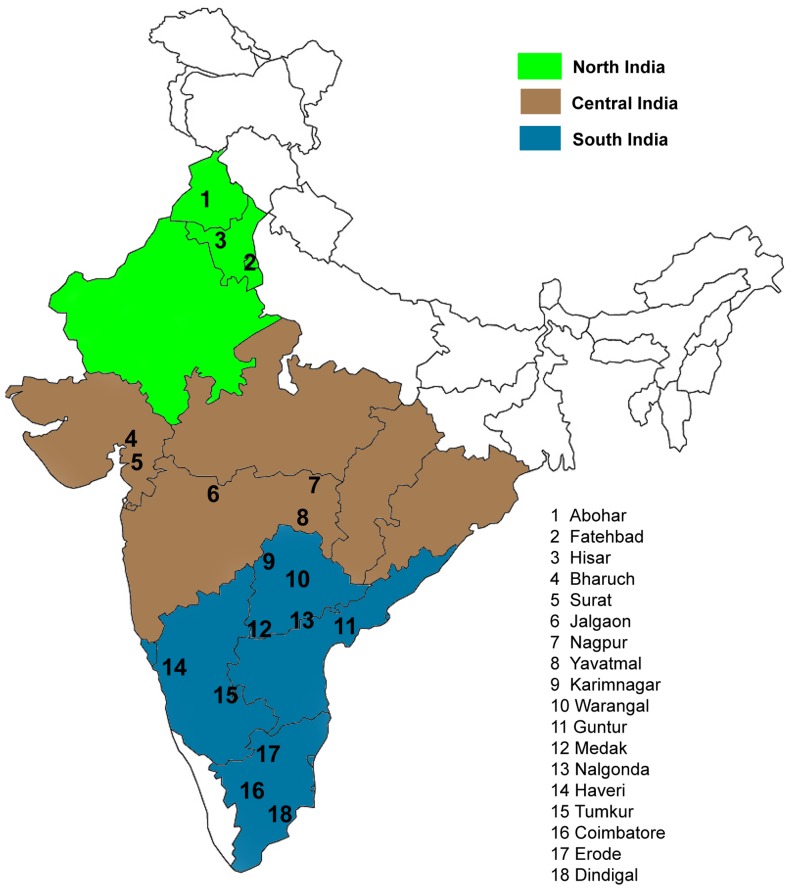
Sampling locations in India.

A total of 786 *H. armigera* individuals were collected from India in the three cropping seasons 2004–5, 2005–6 and 2006–7, as larvae, or moths (Fig. 1 and Table 1). Collections were made from 14 populations on cotton (*Gossypium hirsutum*, Malvaceae), 5 populations of pigeonpea (*Cajanus cajan*, Fabaceae), 4 populations of chickpea (*Cicer arietum*, Fabaceae) and one of eggplant (*Solanum melongena*, Solanaceae). Larvae were collected by direct sampling from different host plants, either directly into ethanol until needed for gDNA extraction, or kept on artificial diet until the pupal stage. Some of these were taken as samples only after they had emerged into adult moths. Male moths from Nagpur_1 and Nagpur_2 were collected by pheromone traps (Table 1). All pupae and adult moth samples were also preserved in absolute ethanol at −20°C until required for DNA extraction. Only a small portion (5 mm of the posterior portion of larvae and pupae, or half the abdomen of adults) of each sample was used for genomic DNA (gDNA) extraction as previously reported [26] or using the method of Zraket et al. [43] with slight modifications. Absence of cross-contamination during the gDNA extraction process was confirmed by the inclusion of a blank extraction among each gDNA extraction batch. The PCR-RFLP (Restriction Fragment Length Polymorphism) *Helicoverpa* species diagnostic test of Behere et al. [44] was used to confirm that all larvae sampled for this study were *H. armigera*.

**Table 1:** Collection details of *H. armigera* populations screened with five EPIC PCR markers.

Regions	Location	n	Latitude	Longitude	Date	Host
Northern India	Abohar_1	15	30°07′N	74°12′E	Jan. 05	Chickpea
	Abohar_2	39	30°07′N	74°12′E	Sept. 06	Cotton
	Hissar	31	29°07′N	75°41′E	Sept. 06	Cotton
	Fatehbad	34	29°30′N	75°27′E	Sept. 06	Cotton
Central India	Bharuch	28	21°42′N	73°00′E	Sept. 05	Cotton†
	Surat	18	21°11′N	72°48′E	Sept. 05	Cotton†
	Jalgaon_1	58	21°01′N	75°33′E	Dec. 05	Cotton†
	Jalgaon_2	44	21°01′N	75°33′E	Jan. 06	Chickpea
	Nagpur_1	18	21°09′N	79°05′E	Jan. 05	Pheromone
	Nagpur_2	40	21°09′N	79°05′E	Sept. 04	Pheromone
	Yavtamal_1	10	20°23′N	78°08′E	Jul. 05	Egg Plant
	Yavtamal_2	23	20°23′N	78°08′E	Oct. 05	Cotton†
	Yavtamal_3	39	20°23′N	78°08′E	Oct. 05	Pigeonpea
	Yavtamal_4	40	20°23′N	78°08′E	Nov. 05	Chickpea
	Yavtamal_3A	31	20°23′N	78°08′E	Nov. 06	Pigeonpea
Southern India	Karimnagar	11	18°26′N	79°08′E	Oct. 05	Cotton
	Warangal	11	18°00′N	79°35′E	Oct. 05	Cotton
	Guntur_1	26	16°17′N	80°25′E	Oct. 05	Cotton
	Guntur_2	42	16°17′N	80°25′E	Dec. 06	Cotton
	Medak	39	18°03′N	78°16′E	Dec. 06	Chickpea
	Nalgonda	30	17°03′N	79°16′E	Dec. 06	Pigeonpea
	Haveri	30	14°47′N	75°24′E	Nov. 06	Pigeonpea
	Tumkur	39	13°20′N	77°04′E	Dec. 06	Cotton
	Coimbatore	22	11°00′N	76°58′E	Jan.05	Pigeonpea
	Erode	34	11°20′N	77°43′E	Jan. 05	Cotton
	Dindigal	34	10°21′N	77°58′E	Dec. 06	Cotton

Numbers after locations represent different collection periods. Known *Bt* cotton hosts are indicated by ‘†’. All insects were collected as mid to late instar larvae except for the Nagpur populations where moths were captured at pheromone traps.

### RpS2 EPIC marker allele characterisation

EPIC-PCR markers RpL3, RpL12, RpL29, RpS6 and RpS2 [42] were utilised to infer population genetic structures in Indian *H. armigera* populations. Molecular characterisation of RpS2 EPIC-PCR marker allele polymorphisms has not been previously reported and is here investigated using the methods described in Tay et al. [42]. Ten RpS2 EPIC maker alleles from Australian and Chinese *H. armigera* individuals (samples previously used for allele characterisation by Tay et al. [42]) were randomly chosen, cloned and sequenced to ascertain the presence of allele homoplasy and nucleotide insertions/deletions (Indels).

### Fluorescence labelling of polymorphic EPIC markers and screening

The forward primer of each EPIC-PCR marker was labelled with a fluorescent dye (FAM, HEX, or TET) to allow detection during electrophoresis. All amplifications were performed in a 15 µL reaction volume consisting of 7.5 µL of 5× GoTaq® Green Master Mix (Promega # M7122); 1.8 mM MgCl_2_; 0.5 µM of each labelled forward and reverse primer and 50–75 ng of template DNA. The PCR amplification profile consisted of an initial template denaturation step of 5 min at 95°C (1 cycle); followed by 35 cycles that consisted of template denaturation (95°C, 1 min) – primer annealing at specific temperature for 1 minute [36] – template extension (72°C, 1 min); and a final 10 min template extension at 72°C (1 cycle). PCR amplicons (5 µL) of all five loci were loaded on 1% ethidium bromide-stained 1× tris-borate-EDTA (TBE) agarose gels, run at 90 V for 90 minutes and visualised over a UV-illuminator, prior to individually multiplexed in 96-well plates by pooling 1 µL of PCR product for each of the loci labelled with three different florescent dyes. For genotyping, DNA fragment sizes were determined by a MEGABASE 1000 automated sequencer (Amersham Biosciences) at the Genetic Analysis Facility (GAF, James Cook University, Queensland, Australia). A size standard (400-R) was co-loaded with every sample to allow accurate sizing of DNA fragments. The final volume was adjusted to 10 µL with dH_2_O; post-PCR desalting was undertaken according to the protocol provided by GAF. Genotypes were scored manually with the help of marker panel set options implemented in the program GENETIC PROFILER 2.2 (Amersham Biosciences). All genotypes were scored unambiguously and where necessary allele peaks were corrected manually. Individuals which amplified for one locus but failed to amplify in PCR for other loci, were repeat amplified for up to a further two times. If a sample failed to amplify across all loci in at least one of the three rounds of PCR, it was considered as a DNA-extraction failure and discarded from subsequent analyses.

### Analysis of null alleles in EPIC-PCR markers

To estimate EPIC-PCR markers null allele frequencies we designed nested (alternative) EPIC-PCR forward and reverse primers for the EPIC-PCR primer pairs used (Table 2). For alt_RpS2 and alt_RpL29 EPIC-PCR primers, we tested 42 randomly selected individuals which were identified as homozygotes using the original RpS2 and RpL29 EPIC markers, as well as six heterozygotes as positive controls. For alt_RpS6, the total number of individuals re-tested was 46 (18 homozygotes, 28 heterozygotes), and for alt_RpL3 a total of 46 individuals (40 homozygotes, 6 heterozygotes) were re-tested. Null alleles were considered detected if during re-genotyping by nested EPIC-PCR markers individuals previously scored as homozygous were found to be heterozygous and vice versa.

**Table 2:** Primer sequences of four nested EPIC PCR markers used for screening *H. armigera* populations for estimating null allele frequencies.

Locus	Primer sequences (5′ to 3′)	Fluorescent dye	Ta (°C)	Exon size difference from Tay *et al.* 2008 [42]	Expected allele size difference
Alt_RpS2	**F** AGAGGTTACTGGGGTAACAAG	TET	50	−5 bp	
	**R** GACACAATACCAGTACCACGAG			−3 bp	8 bp
Alt_RpL29	**F** CAAAGTCAAAGAATCACACAAAT	TET	50	−6 bp	
	**R** GGGTGGATTCGTGCCTTTG			−3 bp	9 pb
Alt_RpS6	**F** CAGGGAGTCCTCACYAAC	TET	50	−20 bp	
	**R** CTTTGACATCARCARACGA			n.a.†	20 bp
Alt_RpL3	**F** GTGTYACMAAGGGYAAAGGAT	FAM	50	−6 bp	
	**R** GTGTGCCAACGGGAGGTCAC			−10 bp	16 bp

† Alt_RpS6 EPIC-PCR marker is 5 nucleotides shorter than that reported [36] however it utilised the first 19 bp of the 24 bp original RpS6 EPIC-PCR oligo.

### Data analysis

Basic statistics for the EPIC-PCR data (average number of alleles per locus, allelic richness averaged over loci, and Weir and Cockerham's measures of *F*_IS_ [45]) were calculated using FSTAT version 2.9.3 [46]. *F*_IS_, an inbreeding coefficient, measures the reduction in heterozygosity of an individual due to non-random mating within its sub-population. Observed (Ho) and expected (He) heterozygosity were estimated and departures from Hardy-Weinberg equilibrium (HWE) were tested using the probability test as implemented in GENEPOP version 3.2 [47]. The tests for genotypic linkage disequilibrium among pairs of loci were performed in GENEPOP using Fisher's tests [47], with unbiased *P* values derived by a Markov chain method (10,000 de-memorisations, 1,000 batches and 10,000 iterations/batch). The significance values for multiple significance tests were set using the sequential Bonferroni procedure [48] within the population genetics software FSTAT. To investigate population differentiation, pair-wise *F*_ST_ estimates [45] (with 95% confidence limits) and significances (determined with 6,500 permutations) were calculated using FSTAT.

The geographic partitioning regime used by Kranthi et al. [49], [50] was followed. Genetic diversity was partitioned into three model structures according to geographic regions (northern, central and southern India; Fig. 1), host crops (cotton, pigeonpea chickpea and egg plant), and cropping seasons (season 2004–05, 2005–06 and 2006–07). Within each model structure, the genetic variation was further partitioned into three levels: (1) among geographic region/host/cropping season; (2) among populations within geographic regions/hosts/cropping seasons, and (3) within populations. A hierarchical analysis of molecular variance (AMOVA) was carried out using pair-wise *F*_ST_ as the genetic distance measure using the population genetics software ARLEQUIN 3.1 [51], [52]. In pair-wise *F*_ST_ estimates and Structure analysis, the two pheromone-trapped populations (Nagpur_1 and Nagpur_2) were excluded because of the unknown host crops. Erode, the only cotton population in season 1 (2004–05), was also excluded from these analyses.

The program Structure v2.3.2 [47] that implements a Bayesian clustering method, was used to identify admixed individuals and for assignment to likely membership of population genetic clusters (‘*K*’) through the assumption of known source populations and HWE at all loci [53]. To estimate the most likely *K* we evaluated all possible *K's* (i.e., *K* = 1 representing no genetic structure, to *K* = 23 representing each population being genetically distinct) using simulation of 20 iterations, with each iteration consisted of 50,000 ‘burnin’ followed by 500,000 Markov Chain Monte Carlo (MCMC) replications, with default settings for both the Ancestry Model (Admixture Model) and the Frequency Model (allele frequencies correlated among populations; assumed different *F*_ST_ values for subpopulations). The Δ*K* method of Evanno et al. [54] was used to ascertain the most likely *K* value, although the log probabilities of data (Ln P(D)) for *K* were also evaluated. The proportions of an individual's genome belonging to particular *K* population clusters are given a ‘*Q*’ score which enables Structure to assign individuals (or portions of an individual's genome) to a particular cluster [53].

## Results

### Null alleles in EPIC-PCR markers

Of the five sets of nested EPIC-PCR markers, we failed to design an alternative RpL12 EPIC primer due to the short exon sequence available, and the null allele frequency for this marker was therefore not estimated. Based on the alternative EPIC-PCR markers alt_RpS2, alt_RpL29, alt_RpL3 and alt_RpS6, null allele frequencies for the original EPIC-PCR markers were estimated at 9.4%, 6.5%, 6.3%, and 4.3% respectively which were considered as being at a moderate level [55], and were within the null allele frequency range (i.e., 2.2%–10.3%) of microsatellite DNA markers used by Endersby et al. [28] for inference of Australian *H. armigera* population genetics structure. As we were unable to estimate the null allele frequency of RpL12, analyses of population substructure patterns of our Indian samples were performed by both including or excluding RpL12 (Table 3). *F*_IS_ estimates from excluding the RpL12 locus remained unchanged for three populations (Abohar_1, Hisar and Karimnagar), reduced in seven populations (Nagpur_2, Yavatmal_2, Yavatmal_3, Warangal, Guntur_1, Nalgonda and Coimbatore), and increased in the remaining 16 populations. Taken as a whole, the inclusion of the RpL12 locus lead to lower and/or no change in *F*_IS_ estimates in 19 of the 26 populations studied, and did not drastically lower *F*_IS_ estimates in cotton populations, suggesting that this marker is unlikely to have harboured an excessively high frequency of null alleles.

**Table 3:** Population statistics based on five EPIC PCR markers tested on Indian *H. armigera* populations.

Regions	Locations	n	a	r	Ho	He	*F*_IS_	Ho*	He*	*F*_IS_ *
Northern India	Abohar_1	15	5.8	4.62	0.36	0.51	0.305	0.45	0.64	**0.305**
	Abohar_2	39	5.2	4.07	0.33	0.59	0.455	0.35	0.69	0.498
	Hisar	31	4.4	3.69	0.24	0.50	0.514	0.31	0.62	**0.514** ^c^
	Fatehbad	34	6.2	4.00	0.18	0.49	0.628	0.21	0.59	0.644
Central India	Bharuch	28	4	3.14	0.36	0.37	0.034	0.44	0.45	0.035
	Surat	18	4.6	3.79	0.35	0.51	0.31	0.39	0.58	0.343
	Jalgaon_1	58	14	5.91	0.35	0.63	0.445	0.40	0.74	0.463
	Jalgaon_2	44	11	5.53	0.36	0.62	0.422	0.40	0.72	0.454
	Nagpur_1	18	7.2	5.32	0.39	0.59	0.333	0.48	0.72	0.340
	Nagpur_2	40	11	5.33	0.37	0.61	0.396	0.43	0.70	0.386
	Yavatmal_1	10	4.4	4.27	0.27	0.52	0.501	0.31	0.63	0.521
	Yavatmal_2	23	8.6	5.45	0.30	0.60	0.513	0.37	0.73	0.499 ^c^
	Yavatmal_3	39	13	6.61	0.37	0.65	0.445	0.42	0.75	0.440
	Yavatmal_4	40	11	5.79	0.35	0.64	0.465	0.39	0.74	0.477
	Yavatmal_3A	31	8.6	5.29	0.30	0.56	0.467	0.35	0.67	0.472
Southern India	Karimnagar	11	4.8	4.52	0.36	0.53	0.319	0.45	0.66	**0.319** ^c^
	Warangal	11	3.6	3.52	0.36	0.54	0.354	0.45	0.68	0.353 ^c^
	Guntur_1	26	8.6	5.33	0.28	0.59	0.521	0.35	0.67	0.492 ^c^
	Guntur_2	42	13	5.99	0.30	0.61	0.517	0.33	0.70	0.537
	Medak	39	10	5.51	0.31	0.60	0.486	0.35	0.72	0.513
	Nalgonda	30	9.4	5.45	0.40	0.59	0.324	0.48	0.70	0.321
	Haveri	30	9.2	5.41	0.38	0.57	0.342	0.45	0.69	0.350
	Tumkur	39	9.2	5.41	0.42	0.62	0.323	0.48	0.71	0.324
	Coimbatore	22	4.8	3.65	0.37	0.50	0.273	0.46	0.61	0.246
	Erode	34	8.8	5.29	0.38	0.55	0.305	0.42	0.63	0.339
	Dindigal	34	9.2	5.29	0.37	0.61	0.396	0.44	0.74	0.408

The number of individuals screened for all five loci is indicated (n); mean values for the number of alleles (a), allelic richness (r), observed heterozygosity (Ho), expected heterozygosity (He), and inbreeding coefficient (*F*_IS_) were also estimated for all populations. Analyses excluding the locus RpL12 are indicated by ‘*’. Cotton populations where *F*_IS_* estimates either remained unchanged (values in bold) or decreased (values underlined) through the exclusion of the RpL12 locus are indicated by ‘c’.

### EPIC marker variability

A total of 155 alleles were scored from the five loci (RpL3, RpL12, RpL29, RpS2 and RpS6) in 26 populations (n = 786) of *H. armigera*. The most polymorphic marker was RpS6 (55 alleles), followed by RpL29 (49 alleles), RpL3 (19 alleles), RpS2 (14 alleles) and RpL12 (15 alleles). The mean number of alleles and the mean observed (*H*o) and expected (*H*e) heterozygosities for each population are shown in Table 3. The average observed heterozygosity value for the five loci was 0.34 (range: 0.18–0.42). Estimates of observed heterozygosity were lower than expected in all populations, and levels of allele richness did not differ significantly between populations. Molecular characterisation of 10 randomly selected alleles (GenBank EU707432–EU707441) from the RpS2 EPIC marker showed no allele homoplasy, with allele length polymorphisms due to Indels within the intron.

### Hardy-Weinberg equilibrium

Departures from Hardy-Weinberg equilibrium over all loci were found in all populations of *H. armigera*. This significant deviation from Hardy-Weinberg equilibrium was due to an excess of homozygotes at all loci, and is further reflected by the *F*_IS_ values (Table 3). Genotypic linkage disequilibrium tests found no significant associations between pairs of loci for any populations or over all populations after Bonferroni corrections for multiple comparisons, indicating independent assortment for these Rp EPIC markers.

### Analysis of Molecular Variance (AMOVA) and *F* statistics

AMOVA analysis (Table 4) indicated that as a whole, Indian populations showed similar levels of genetic variability regardless of geographic regions (−0.06%, *F*_CT_ = −0.006, *P* = 0.36), host plants (−0.68%, *F*_CT_ = −0.007, *P* = 0.98) or cropping seasons (−0.23%, *F*_CT_ = −0.002, *P* = 0.65). However significant genetic structures were detected among populations within geographic regions (4.51%, *F*_SC_ = 0.045, *P*<0.001), within host plants (i.e., within host plant species across sampling sites) (5.07%, *F*_SC_ = 0.050, *P*<0.001) and within cropping seasons (4.63%, *F*_SC_ = 0.046, *P*<0.001). Large variations (*F*_ST_>95%) were found within populations in each of three model structures (geographic region, host and cropping season; Table 4). To better understand underlying factors that contributed to within population genetic variations, data from two cropping seasons (2005–06 and 2006–07) were partitioned individually according to geographic region or plant host. The overall trend remained similar (i.e. there remained significant variation between populations within geographic regions and within hosts (data not shown)). Therefore, values for these significant genetic variations were examined in detail by estimates of pair-wise *F*_ST_ values. The overall pair-wise *F*_ST_ values ranged from −0.001 to 0.431, with higher *F*_ST_ values in general being associated with cotton populations (Table 5). Cotton populations collected in the 2006–07 cropping season further differed significantly between northern and southern India (Table 5). Within cotton, the highest *F*_ST_ values were seen in the Bharuch population when compared with all other populations. Pair-wise *F*_ST_ values for populations from food crops (pigeonpea, chickpea and eggplant) contrasted with those observed in cotton populations and were generally non-significant (except Coimbatore) (range: −0.008 to 0.100; Table 6).

**Table 4:** Comparisons of genetic variation by AMOVA based on data generated by EPIC-PCR markers from *Helicoverpa armigera* according to three population structure hierarchical models.

Model	Hierarchical levels	Degrees of freedom	Sum of square	Variance components	Fixation indices	Percentage of variation	*P*- value
*Model A Geographic regions*	Among regions	2	10.849	−0.0009	−0.0062 *F*_CT_	−0.06	0.36
	Among populations within regions	23	122.464	0.0660	0.0451 *F*_SC_	4.51	<0.001
	Within populations	1546	2162.19	1.3986	0.0445 *F*_ST_	95.55	<0.001
	Total	1571	2295.50	1.4637			
*Model B Hosts*	Among Hosts (cotton, pigeonpea, chickpea)	2	5.17	−0.0099	−0.007 *F*_CT_	−0.68	0.98
	Among populations within host crop	20	118.48	0.0739	0.0504 *F*_SC_	5.07	<0.001
	Within populations	1413	1967.81	1.3927	0.0440 *F*_ST_	95.60	<0.001
	Total	1435	2091.81	1.4567			
*Model C Cropping seasons*	Among seasons	2	8.41	−0.0034	−0.0023 *F*_CT_	−0.23	0.65
	Among populations within seasons	23	124.90	0.0677	0.0462 *F*_SC_	4.63	<0.001
	Within populations	1546	2162.19	1.3986	0.0439 *F*_ST_	95.61	<0.001
	Total	1571	2295.50	1.4628			

Model A: compares variation between geographic regions (northern India, central India, and southern India), among populations within regions and within populations. Model B: compares variation between hosts (cotton, pigeonpea and chickpea), among populations within hosts and within populations. Model C: compares genetic variations between cropping seasons (season 2004–05, season 2005–06, season 2006–07), among populations within cropping seasons and within populations.

**Table 5:** Pair-wise *F*_ST_ values (below diagonal) and associated *P*-values (above diagonal) for *H. armigera* populations collected from cotton during cropping seasons 2 (2005–2006) and 3 (2006–2007).

Region			CI	CI	CI	CI	SI	SI	SI	NI	NI	NI	SI	SI	SI
	Cropping Season		2	2	2	2	2	2	2	3	3	3	3	3	3
		Sampling locations	Bharuch	Surat	Yavatmal_2	Jalgaon_1	Karimnagar	Warangal	Guntur_1	Abohar_2	Hissar	Fatehbad	Guntur_2	Tumkur	Dindigal
CI	2	Bharuch	-	0.000	0.000	0.000	0.000	0.000	0.000	0.000	0.000	0.000	0.000	0.000	0.000
CI	2	Surat	**0.192**	-	0.001	0.000	0.000	0.001	0.002	0.000	0.000	0.000	0.005	0.000	0.001
CI	2	Yavatmal_2	**0.275**	0.061	-	0.188	0.007	0.002	0.741	0.000	0.000	0.004	0.030	0.000	0.031
CI	2	Jalgaon_1	**0.142**	**0.033**	0.007	-	0.000	0.074	0.233	0.000	0.000	0.000	0.638	0.000	0.396
SI	2	Karimnagar	**0.431**	**0.202**	0.052	**0.092**	-	0.010	0.003	0.000	0.000	0.000	0.001	0.000	0.002
SI	2	Warangal	**0.244**	0.097	0.054	0.020	0.074	-	0.003	0.000	0.000	0.000	0.007	0.001	0.001
SI	2	Guntur_1	**0.228**	0.033	−0.007	0.003	0.112	0.060	-	0.000	0.000	0.000	0.174	0.000	0.012
NI	3	Abohar_2	**0.240**	**0.084**	**0.014**	**0.033**	**0.065**	**0.065**	**0.037**	-	0.000	0.000	0.000	0.000	0.000
NI	3	Hissar	**0.200**	**0.041**	**0.048**	**0.025**	**0.140**	**0.075**	**0.035**	**0.074**	-	0.000	0.000	0.000	0.000
NI	3	Fatehbad	**0.247**	**0.055**	0.044	**0.038**	**0.103**	**0.055**	**0.045**	**0.060**	**0.017**	-	0.000	0.000	0.000
SI	3	Guntur_2	**0.116**	0.028	0.031	−0.002	0.128	0.043	0.016	**0.044**	**0.036**	**0.052**	-	0.000	0.450
SI	3	Tumkur	**0.141**	**0.044**	**0.042**	**0.016**	**0.087**	0.030	**0.040**	**0.048**	**0.040**	**0.034**	**0.013**	-	0.000
SI	3	Dindigal	**0.164**	0.042	0.008	−0.001	0.073	0.033	0.015	**0.019**	**0.038**	**0.039**	0.003	**0.012**	-

*P-*values obtained after 1,560 permutations. Values in bold are significant at *P*<0.00064 after sequential Bonferroni correction. Geographic regions are northern India (NI), central India (CI), and southern India (SI).

**Table 6:** Pair-wise *F*_ST_ values (below diagonal) and associated *P*-values (above diagonal, values obtained from 720 permutations) for *Helicoverpa armigera* populations collected from food crops (eggplant (ep), chickpea (cp) and pigeonpea (pp)).

Crops and regions			ep (CI)	cp (NI)	pp (SI)	cp (CI)	pp (CI)	cp (CI)	pp (CI)	cp (SI)	pp (SI)	pp (SI)
	Cropping season		1	1	1	2	2	2	3	3	3	3
		Sampling locations	Yavatmal_1	Abohar_1	Coimbatore	Jaglaon_2	Yavatmal_3	Yavatmal_4	Yavatmal_3A	Medak	Nalgonda	Haveri
ep (CI)	1	Yavatmal_1	-	0.037	0.000	0.001	0.262	0.006	0.026	0.029	0.007	0.062
cp (NI)	1	Abohar_1	0.041	-	0.003	0.064	0.243	0.008	0.392	0.014	0.410	0.649
pp (SI)	1	Coimbatore	0.148	0.069	-	0.001	0.001	0.011	0.003	0.004	0.001	0.001
cp (CI)	2	Jaglaon_2	0.038	0.006	**0.066**	-	0.007	0.003	0.075	0.003	0.069	0.114
pp (CI)	2	Yavatmal-3	0.018	0.022	**0.086**	0.006	-	0.013	0.040	0.001	0.001	0.197
cp (CI)	2	Yavatmal_4	0.048	0.016	0.029	0.004	0.009	-	0.086	0.006	0.011	0.011
pp (CI)	3	Yavatmal_3A	0.032	−0.008	0.058	−0.002	0.009	0.003	-	0.022	0.474	0.339
cp (SI)	3	Medak	0.066	0.037	0.042	0.021	**0.023**	0.006	0.019	-	0.003	0.001
pp (SI)	3	Nalgonda	0.041	−0.006	**0.074**	0.001	**0.018**	0.009	−0.005	0.019	-	0.350
pp (SI)	3	Haveri	0.031	0.000	**0.101**	0.007	0.010	0.018	0.012	**0.047**	0.006	-

Values in bold are significant at *P*<0.0013 after sequential Bonferroni correction; sampling regions are (NI) northern India, (CI) central India, and (SI) southern India.

### Structure Analyses

**Figure 2:**
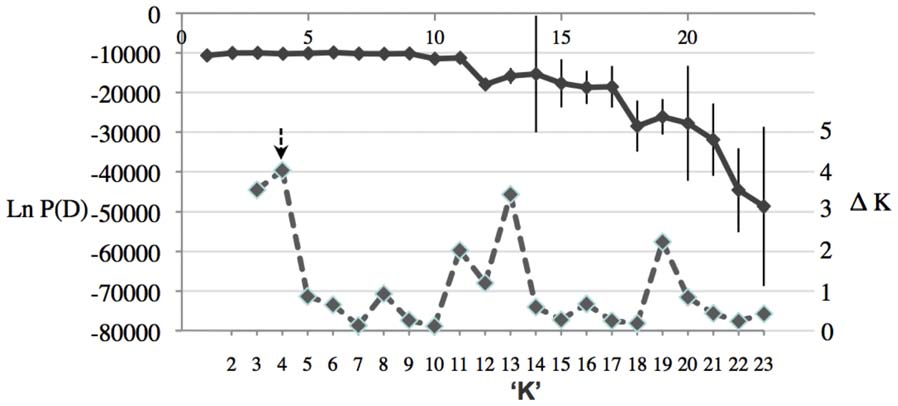
Structure analysis of 23 *Helicoverpa armigera* populations collected from India from cotton crop (years 2 and 3) and food crops (years 1, 2, 3). Locations and populations are as in Fig. 1 and Table 1: northern India (1) Abohar, (2) Fatehbad, (3) Hissar; central India (4) Bharuch, (5) Surat, (6) Jalgaon, (8) Yavatmal; southern India (9) Karimnagar, (10) Warangal, (11) Guntur, (12) Medak, (13) Nalgonda, (14) Haveri, (15) Tumkur, (16) Coimbatore, and (18) Dindigal. Average log likelihood of data Ln P(D) (primary axis, solid line) and Δ*K* (secondary axis, dashed line) are shown, with best K = 4 indicated an arrow.

**Figure 3:**
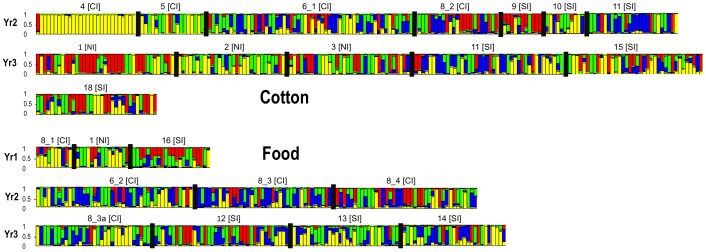
*Helicoverpa armigera* population structure as inferred using the Bayesian clustering algorithm implemented in the program Structure 2.3. Bar graph of *K* = 4 for all individuals from 23 populations collected across three sampling years from either cotton or food crop plant hosts are shown. Locations and populations are as in Fig. 1 and Table 1, Indian agricultural regions are northern Indian (NI), central India (CI) and southern Indian (SI).

**Figure 4:**
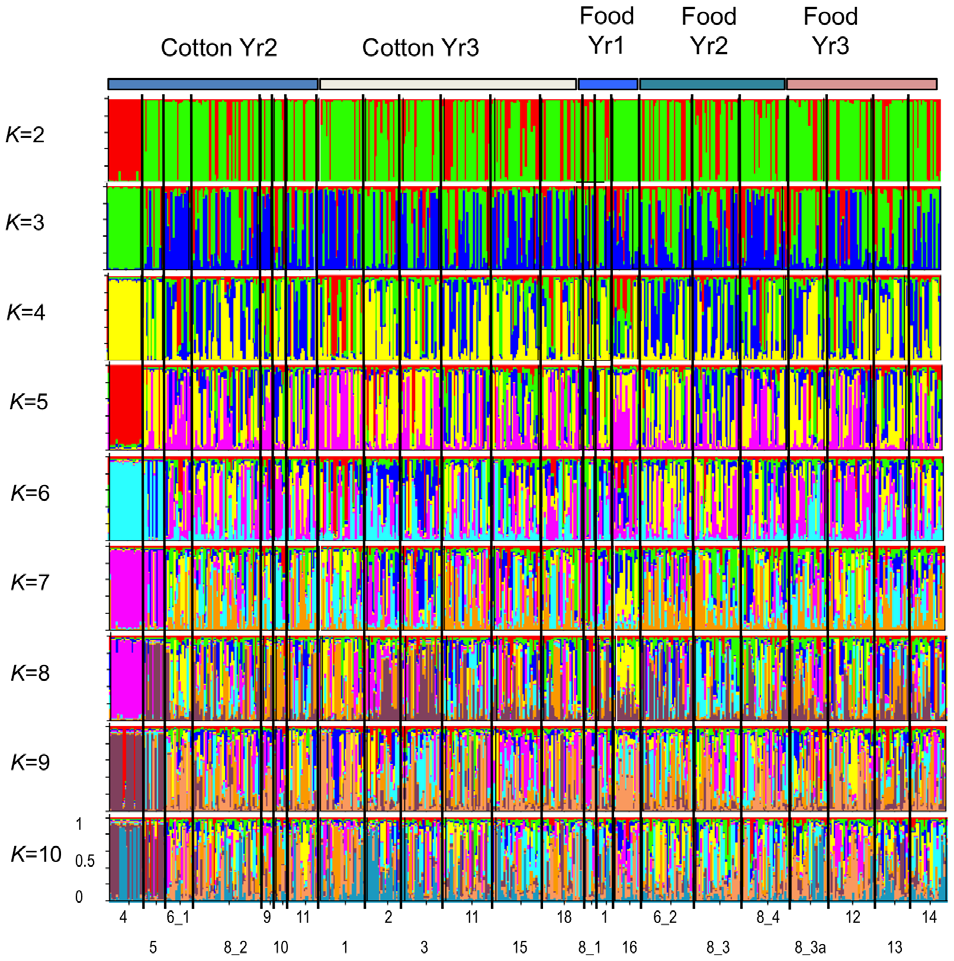
Population structure of 23 *H. armigera* populations inferred using the Structure program after 50,000 ‘burnin’ followed by 500,000 MCMC replications. Each individual is represented by a single line, with black lines separating between populations. The estimated membership fractions for K = 2 to K = 10 clusters for all individuals are shown.

Based on the averaged Ln P(D) of 20 simulations for each *K* = 1 to *K* = 23, the best *K* was identified as *K* = 8 (−9912.76±536.43 s.d.). However, using the Evanno et al. [54] method identified the best Δ*K* value (614.37) at *K* = 2 followed by the second highest Δ*K* value of 4.06 at *K* = 4 (Fig. 2, Fig. 3). Although the Δ*K* value was largest for *K* = 2, this large change probably indicated a shift from the unlikely scenario of our samples showing no structure (i.e., *K* = 1) towards more plausible scenario of presence of population substructure (i.e., *K*>1). The large and positive Δ*K* value for *K* = 4 (Fig. 2) was therefore selected as the cluster number most likely to assist in visualising significant population substructure. The generalised patterns of population structure for *K* = 2 to *K* = 10 are presented (Fig. 4) to help visualise the selection of K = 4. A detailed Structure bar graph at K = 4 across all 23 populations was presented (Fig. 3). No obvious biologically relevant population structure patterns could be inferred from the Structure analysis, and setting of *K*>4 clusters progressively introduced greater population heterogeneity and further reduced the power of interpretation, even for the Ln P(D) optimum *K* = 8. Between *K* = 2 and *K* = 9, the Bharuch (pop. 4) and Surat (pop. 5) populations in central India appeared highly homogeneous, while all other populations showed higher levels of admixture (Fig. 4). Detailed examination of the Structure analysis at *K* = 4 (Fig. 3) showed that substantial substructure within and between populations existed, with populations which were geographically close to each other sorting into very different genetic clusters. Across the three sampling years (Fig. 3), reduced genetic diversity was seen only in some cotton populations (e.g., Year 2: Baruch (pop. 4), Surat (pop. 5), Karimnagar (pop. 9); Year 3: Abohar (pop. 1), Fatehebad (pop. 2)). Substantial substructure between populations can be seen, for example, in Year 2 cotton populations between Baruch (pop. 4)/Surat (pop. 5) and Guntur (pop 11)/Karimnagar (pop. 9)/Yvatmal (pop. 8). Substructure of populations is further seen between populations which are in close geographic proximity (e.g. in cotton Year 3: Abohar (pop. 1), Fatehbad (pop. 2) and Hissar (pop. 3); food crops Year 3: Medak (pop. 12) and Nalgonda (pop. 13)). This contrasted with the genetic patterns of food crop populations (Table 6), which did not show such strong substructure.

## Discussion

### *H. armigera* population genetics inferred from EPIC markers

Population genetic analysis of Indian *H. armigera* samples using EPIC markers clearly indicated no obvious population substructure with geographic region, year or crop, and indicated significant genetic differentiation between northern and central/southern Indian cotton-feeding populations in the cropping season of 2006–07. Within the populations HW non-equilibrium was detected, with a likely contributing factor being the presence of null alleles within our DNA marker system. Null allele frequency estimates for four of these EPIC-PCR markers were moderate (4.3% to 9.4%). The conclusion on population substructure patterns remained overall unaltered regardless of the inclusion or exclusion of the untested RpL12 locus from analysis. In the population genetic study involving predominantly Australian *H. armigera* populations, Endersby et al. [28] excluded 3 pairs of SSR markers with the greatest null allele frequencies (i.e., 19.2%, 31.6% and 47.4%), and demonstrated that DNA markers with low to moderate levels of null allele frequencies were sufficiently powerful to enable meaningful interpretation of gene flow patterns, a conclusion consistent with population genetics simulation studies [55], [56]. Chapuis and Estoup [55] also concluded that the most accurate *F*_ST_ estimates were obtained when null alleles in marker systems were not excluded (i.e. by scoring only visible alleles within study populations), although within populations with substantial gene flow (i.e., non-cotton populations in this study), *F*_ST_ estimates were less biased than in populations with limited gene flow (i.e., cotton populations).

Our study further demonstrated that null allele events in *H. armigera* population genetic markers are relatively common, despite attempts to minimise their occurrence by designing markers at conserved gene coding regions. The effort in designing EPIC-PCR markers has other advantages such as enabling nested PCR markers to be developed for retesting of populations at the same loci, as well as the overall lower null allele frequencies as compared with null allele frequencies from random SSR markers which may be associated with TE's [39], [57]. Null alleles or inbreeding may also contribute to heterozygote deficiency. A locus-specific heterozygote deficit is an indication of null alleles, rather than inbreeding or other population processes which are generally reflected across all loci. Significant heterozygote deficiencies were detected at all five loci in most *H. armigera* populations tested, further indicating only low to moderate levels of null alleles affecting our EPIC markers.

A Wahlund effect (i.e., fine-scale heterogeneity versus large-scale homogeneity; e.g., see [58]) as indicated in our high *F*_IS_, may further affect interpretations of population structure of *H. armigera* in India. The sample size of n = 786 allowed us to detect significant departures from HWE for all five EPIC markers. Although the Structure analysis assumed that all potential source populations were sampled when assigning genetic clusters to individuals, our study was aimed at understanding broader *H. armigera* population patterns in Indian's highly heterogeneous agricultural landscape rather than inferring individuals' specific origins. The lack of interpretable and biologically meaningful Structure results as well as the general patterns of low population substructure in our study could potentially be due to our markers being in non-HWE (i.e., Structure analysis assumes HWE in loci, [53]), although various authors (e.g., De Barro [59], Brown et al. [60]) reported no apparent significant effects to Structure results in loci that did not demonstrate HWE. Our finding that the Bharuch and Surat populations are generally highly homogeneous may therefore either be an indication of a lack of gene flow and/or due to selection.

Departures from HWE due to homozygous excess may represent true biological phenomena in *H. armigera* such as those due to strong inbreeding caused by frequent bottlenecks, the Wahlund effect (e.g., Nielsen et al. [61]), or be due to extrinsic factors such as insecticide selection pressure, *Bt* proteins and/or plant secondary chemicals, or other environmental selectors. In addition to possible effects of utilising microsatellite DNA families/TE-associated loci [57], [39], the generally small-scale heterogeneous Indian cropping landscape, intense selection pressure from heavy insecticide applications, exposure to *Bt* toxins from GM cotton, and/or exposure to host plant secondary compounds could cause *H. armigera* populations to deviate from HWE (although we should note that the later two factors may be similar in Australia).

The different cropping systems to which *H. armigera* is exposed to may also be important underlying factors that contributed to population substructure differences. Host crops with short flowering periods (e.g., food crops such as eggplant, chickpea and pigeonpea) generally support no more than one or two *H. armigera* generation; while hosts such as cotton with prolonged flowering periods are capable of supporting≥three consecutive generations [5], [3]. Populations that feed on cotton are under tremendous selection pressure from insecticide applications [49], and from varying levels of the allelochemical gossypol associated with different life stages and specific cotton varieties [62]. Although host crop species are generally the same across India, the temporal pattern of availability and of the size of the *H. armigera* populations varies greatly, with relatively small populations on only one or a few crops at some times of year in some regions. In northern India, *H. armigera* is known to occur initially on food crops (chickpea, sunflower, some vegetable crops) during February to July prior to feeding on cotton from August (see [63]). A large proportion of the population in the north may enter diapause, to emerge after the partial break between cropping seasons, and re-mix with the smaller, non-diapause population which has been subsisting for 1–2 generations on other, less intensively managed, hosts. The switch from food crops (i.e., with low insecticide exposure) each typically supporting a single *H. armigera* generation, to cotton hosts with increased insecticide and *Bt* toxin exposure capable of supporting multiple generations, can lead to intense selection on sedentary cotton populations. These cotton populations are accompanied by an increase in population densities, with peak infestations typically recorded during September to November in the north (see [63]). These peaks are accompanied by a significant increase in population density in non-GM cotton.

In contrast, central and southern Indian *H. armigera* populations initially feed on cotton (August to October in central India, September to December in southern India) prior to switching over to food crops. Central and southern Indian *H. armigera* populations are therefore likely to experience less consistent insecticide/*Bt* protein selection pressure approaching the end of each cropping season as host crops change from cotton to food crops. This may promote gene exchange between populations as they move between crops. This scenario is the reverse of that in northern India, where populations sampled near the end of the cropping season might be expected to show more differentiation, as selection by insecticides and *Bt* toxins in cotton on such populations potentially operates over several generations with reduced migration, creating a mosaic of genetically different populations across the various cotton types and management systems. This may be part of the explanation for the significant pairwise *F*_ST_ values from cotton populations (especially in northern India) (Table 5) suggesting raised levels of substructures in cotton populations.

### *H. armigera* population structure on cotton

Population genetic analysis of Indian *H. armigera* populations showed a high degree of differentiation between collections within cropping seasons. Based on *F*_ST_ values the observed genetic variation was most marked with populations collected from cotton in cropping season 3 (2006–07). The genetic differentiation revealed by pair-wise estimates of *F*_ST_ values suggested that there existed seasonal and geographical variation within *H. armigera* populations collected from cotton (Table 5). Further, populations from northern India were significantly different from southern Indian populations in cropping season 3, although the underlying factors responsible for this remain unclear.

Overall estimates of pairwise *F*_ST_ values for cotton populations in cropping season 2 between central and southern India were non-significant (Table 5). The exception being the Bharuch population which showed significant *F*_ST_ values against all other populations. The distance between Bharuch and Surat is only 60 km and there are no geographical barriers, differences in cropping practices, or climatic conditions between these areas. Nevertheless these populations were significantly different from each other (*F*_ST_ = 0.192). Possible reasons contributing to such strong genetic differentiation may include different *H. armigera* generations being collected (see Table 1 for collection dates), and/or sampled populations feeding on different cotton types (e.g., *Bt* or non-*Bt* cotton; cotton varieties with different levels of gossypol contents). It is also possible that some of the diversity/structure observed may be associated with the mosaic of *Bt* versus non-*Bt* fields, and this would warrant further study. Pairwise *F*_ST_ values between seasons 2 and 3 in the cotton crop broadly indicated population substructure differences between northern and central/southern Indian, and may reflect underlying differences in cropping patterns (i.e., ‘cotton first’ or ‘cotton last’ in the cropping season).

Fluctuations in host availability may influence *H. armigera* populations and could result in genetic differentiation among local populations, but this assumption is only valid when there is substantial genetic isolation between populations. In India, *H. armigera* population substructure has been further suggested based on feeding preferences [14], insecticide resistance [16], differential response to pheromones [21] and to parasitoids [64]. The abundance, movement and distribution of *H. armigera* were found to be associated with rainfall and humidity in Australia (e.g., [65]) and suggested for India [66]. *H. armigera* is a facultative migrant [1], responding largely to local environmental conditions and host availability (i.e., moths remain sedentary where food resources such as flowering plant hosts are available). Cropping and landscape patterns, as well as insecticide application practices and resistance pest management strategies in *Bt* cotton differ greatly between Australia and India, which therefore limits meaningful comparison between findings from this study and Australian *H. armigera* population genetic structure. For example, the agricultural landscape in India is typically of low acreage, highly diverse and fragmented in the pattern of crop hosts growing at any given time. As such, it generally enables the presence of more than five alternate hosts of *H. armigera* at any given time of the cropping season [2], [67], while there would frequently be only a single major host over the corresponding period in Australia across comparatively large cropping areas within each production region.

### *H. armigera* population structure on food crops

Pigeonpea is cultivated all over India, where it is commonly grown alongside cotton or cultivated as an inter-crop within the cotton agro-ecosystem [68]. Furthermore, flowering periods of cotton and pigeonpea overlap which may facilitate population movements between cotton and pigeonpea, and may explain the high pairwise *F*_ST_ values associated with pigeonpea (Table 6). Pulse crops (i.e., pigeonpea and chickpea) are preferred hosts of *H. armigera* compared to cotton and are planted on larger areas than cotton (Directorate of Economics and Statistics, Department of Agriculture and Cooperation, Ministry of Agriculture, Government of India [69], [70], [71]). Pesticide applications on food crops are, however, less intense than on cotton and *H. armigera* populations are therefore expected to experience less selection for specific genotypes, which may result in a lower level of apparent genetic sub-structure than is seen in cotton. Overall estimates of pairwise *F*_ST_ values among food crops were by and large non-significant in all three cropping seasons (Table 6), although within cropping seasons the number of populations sampled was relatively low.

If the ideas presented above are correct, patterns of population structure analysed in one season should be reflected in the analysis of subsequent seasons. Consistent patterns observed between seasons, hosts and regions would thus support there being host- and/or region-associated micro-population structuring in Indian *H. armigera*. Such patterns have not been clearly seen in this study. Unsurprisingly, the situation is dynamic. For example population 8_3 was collected from Yvatmal on pigeonpea in years 2 and 3 (pop. 8_3a) and shows some shift of genetic profile (Fig. 3). A similar situation exists with cotton populations from Guntur (pop. 11) in years 2 and 3. Although the overall number of Indian populations sampled in this study is comparable to that in other lepidopteran population genetic studies (e.g., *H. armigera*, [28]; *P. xylostella*, [72]; *C. pomonella*, [73]), the complexity of Indian cropping systems nevertheless means that additional populations from northern, central and southern India over the season and on various hosts will be needed to enable more detailed interpretation. *H. armigera* populations analysed in this study do not cover all desired sampling locations and hosts for all seasons, and the patterns seen may also be influenced by factors such as sampling errors. In order to explain this intra-seasonal or host crop-associated genetic differentiation, further analysis of samples collected several times from the same host crops and sites over multiple cropping seasons will be needed.

Understanding the observed genetic structuring in Indian *H. armigera* populations from cotton may be further advanced with research on the detoxification capabilities and ecological aspects of this highly polyphagous pest insect species. Variables such as insecticide usage, *Bt*/non-*Bt* cotton, different hybrids of cotton, different hosts, climatic conditions, and diapause should be considered separately to better ascertain their importance to the population genetic structure of cotton-feeding *H. armigera* in India.
